# The effects of mindfulness‐based interventions on emotion regulation/dysregulation in people with mental health conditions: A systematic review and meta‐analysis

**DOI:** 10.1002/jcv2.70103

**Published:** 2026-02-12

**Authors:** Thomas Easdale‐Cheele, George Nash, Veronika Filobokova, Chloe Westbury, Alessio Bellato

**Affiliations:** ^1^ School of Psychology University of Southampton Southampton UK; ^2^ Developmental Evidence synthesis, Prediction, Implementation (EPI) Lab University of Southampton Southampton UK; ^3^ Department of Psychology University of Roehampton London UK; ^4^ Child and Adolescent Mental Health Service Hampshire and Isle of Wight Healthcare NHS Foundation Trust Southampton UK; ^5^ Centre for Innovation in Mental Health University of Southampton Southampton UK; ^6^ Institute for Life Sciences University of Southampton Southampton UK; ^7^ School of Psychology University of Nottingham Semenyih Malaysia; ^8^ South‐East Asia Mental Health Consortium (SEAMHC) University of Nottingham Semenyih Malaysia

**Keywords:** clinical populations, emotion dysregulation, emotion regulation, mental health treatment, mindfulness‐based interventions, transdiagnostic approaches

## Abstract

**Background:**

We conducted a systematic review and meta‐analysis to examine the effects of mindfulness‐based interventions (MBIs) on emotion regulation (ER) and emotion dysregulation (ED) in people with any mental health condition.

**Methods:**

Following a pre‐registered protocol (PROSPERO CRD42024618605), we searched multiple databases (Web of Science, PsycINFO, Embase, and PubMed) on 04/07/2025. We identified randomised‐controlled trials (RCTs) in which the effects of MBIs on ER or ED were measured in people with mental health conditions established by an adequately trained healthcare professional according to the *Diagnostic and Statistical Manual of Mental Disorders* (from third to fifth editions) or equivalent diagnosis as per the *International Classification of Diseases* (ninth or 10th revisions). Pooled effect sizes (Hedge's *g*) were estimated using random‐effect meta‐analyses. Study quality was assessed using the *Cochrane Risk of Bias Tool 2*.

**Results:**

We identified 19 RCTs, with 16 in the meta‐analyses (988 participants in total; 50.71% randomised to MBIs). We found that MBIs significantly improved cognitive reappraisal (*k* = 6, *g* = 0.65, 95% CI = 0.33, 0.98) and reduced overall ED (*k* = 9; *g* = −0.54; CI = −0.71, −0.36). Significant reductions in ED domains concerning goal‐directedness, impulsivity, and accessing ER strategies were found. Effects for expressive suppression were nonsignificant (*k* = 6; *g* = −0.25; CI = −0.94, 0.45) with significant heterogeneity. Study quality significantly moderated both ER outcomes, though not overall ED.

**Conclusion:**

MBIs show potential for improving cognitive reappraisal and reducing ED across diagnoses. However, limited evidence for younger people and self‐report measurements warrant cautious interpretation.

**Trial Registration:**

NIHR PROSPERO 2024 CRD42024618605. https://www.crd.york.ac.uk/PROSPERO/view/CRD42024618605.

## Key Points

**What's known?**

- Mental health conditions are widespread and often involve shared, transdiagnostic mechanisms that require treatment, such as difficulties with emotion regulation/dysregulation.

**What's new?**

- The present evidence synthesis shows that mindfulness‐based interventions improve cognitive reappraisal and reduce emotion dysregulation across a range of mental health conditions.

**What's relevant?**

- Mindfulness‐based interventions represent a transdiagnostic and broadly accessible option when targeting emotion regulation/dysregulation in people with mental health conditions.

## INTRODUCTION

Mental health conditions are widespread, affecting between 15.7% and 22% of the population (NHS England, 2022; Stansfeld et al., 2016). This prevalence highlights the need for effective, accessible, and resource‐efficient interventions. Diagnosis‐specific interventions face three key limitations: high comorbidity rates (Dalgleish et al., 2020: “Comorbidity … is the rule rather than the exception”.), considerable within‐diagnosis heterogeneity, and considerable phenotypic plasticity of mental health problems (Dalgleish et al., 2020). These limitations have spurred interest in *transdiagnostic approaches* that target shared mechanisms across conditions, rather than diagnostic categories (Dalgleish et al., 2020).

*Mindfulness‐based interventions* (MBIs) show promise as transdiagnostic treatment options. Evidence supports the benefits of MBIs across a wide range of symptoms and conditions, including depression, anxiety, eating disorders, and psychosis (Chayadi et al., 2022; Dunning et al., 2019; Goldberg et al., 2018; Zuo et al., 2023). Specifically, multiple meta‐analyses have reported significant beneficial effects of MBIs for symptoms of anxiety and depression (Gál et al., 2021; Galante et al., 2021; Querstret et al., 2020; Sevilla‐Llewellyn‐Jones et al., 2018; Spijkerman et al., 2016; Witarto et al., 2022), though these effects do differ across disorders. Importantly, robust evidence syntheses examining the effects of MBIs on clinically relevant, transdiagnostic outcomes beyond anxiety and depression remain scarce.

MBIs, such as mindfulness‐based stress reduction (MBSR) and mindfulness‐based cognitive therapy (MBCT), are usually structured, group‐based, instructor‐led, and manualised programmes that combine formal and informal mindfulness practices (Shapero et al., 2018). *Mindfulness* refers to the self‐regulated, non‐judgemental, non‐reactive, and accepting awareness of immediate experience(s) (Eberth & Sedlmeier, 2012; Rau & Williams, 2016; Teper et al., 2013). Though rooted in Buddhist traditions, contemporary MBIs are typically secular and often integrate cognitive‐behavioural elements (Shapero et al., 2018).

Rather than targeting specific conditions, MBIs usually focus on improving broad capacities, such as non‐reactivity, acceptance, and meta‐cognitive awareness, that benefit a range of mental health and transdiagnostic processes, such as *emotion regulation* (ER) and *emotion dysregulation* (ED) (Dunning et al., 2019). This study focuses on the effects of MBIs on ER and ED in clinical populations.

ER refers to the strategies used to attend and respond to emotional experiences in goal‐directed and context‐appropriate ways (D’Agostino et al., 2017; Easdale‐Cheele et al., 2024). Gross' (2002) *Process Model of ER* outlines five ER strategy families: situation selection (i.e., approaching or avoiding specific situations); situation modification; attentional deployment; cognitive change (i.e., reappraisal); and response modulation (i.e., influencing elicited emotion via responses). Flexibility in selecting and applying a wide range of strategies is considered beneficial across conditions (Menefee et al., 2022).

ED is not simply the absence of ER but a multidimensional construct (i.e., cognitive, emotional, behavioural, physiological), reflecting a cause and outcome of ER difficulties (D’Agostino et al., 2017; Easdale‐Cheele et al., 2024). ED is marked by reduced emotional awareness and acceptance, and heightened emotional sensitivity, irritability, and variability (D’Agostino et al., 2017; Easdale‐Cheele et al., 2024). Consequently, ED impairs ER strategy selection and implementation, linked to dysfunctional and disproportionate responses to emotional experiences (D’Agostino et al., 2017; Easdale‐Cheele et al., 2024).

MBIs may improve ER and ED through key mechanisms of mindfulness including increased non‐reactivity, acceptance, and meta‐cognitive awareness (or ‘decentring’), which is the ability to observe emotions and thoughts without becoming entangled in them (Iani et al., 2019; Menefee et al., 2022; Shapero et al., 2018). These mechanisms can promote greater emotional awareness and support both access to and the deliberate use of ER strategies (i.e., their application), though not necessarily enhance strategy quality or repertoire.

As transdiagnostic treatment models gain interest, so does the need to evaluate candidate interventions. Given the theoretical and empirical links between MBIs, ER, and ED, this evidence synthesis study aims, to our knowledge, to be the first to examine the effects of MBIs on ER and ED across mental health conditions and ages, thus maximising our assessment of its transdiagnostic potential. Whilst some variation in responses to MBIs might be seen—or expected—across diagnoses (with important clinical implications), we hypothesise that MBIs will significantly improve ER and reduce ED across mental health conditions.

## METHOD AND MATERIALS

### Search strategy and selection criteria

We followed the most recent Preferred Reporting Items for Systematic Reviews and Meta‐Analyses (PRISMA) guidelines (PRISMA Checklist reported in Supporting Information S1: Appendix S1; Page et al., 2021). Our study was pre‐registered on PROSPERO (CRD42024618605), and we fully adhered to Version 1.1 for research question 1a. We searched Web of Science, PsycINFO, Embase, and PubMed for suitable studies from database inception through July 4, 2025, in line with our main research question.

The search strategy (see Supporting Information S1: Appendix S2) included terms associated with (a) mindfulness and MBIs and (b) ER and ED. Studies were considered eligible for inclusion if they met the following criteria: (1) including people with a diagnosis of any mental health condition (psychological, psychiatric, neurodevelopmental) established by an adequately trained healthcare professional (e.g., psychiatrist or clinical psychologist) according to the Diagnostic and Statistical Manual of Mental Disorders (from third to fifth editions) or equivalent diagnosis as per the International Classification of Diseases (ninth or 10th revision); (2) original, randomised‐controlled trial (RCT) involving the pre‐defined MBIs (MBSR, MBCT, or mindfulness training, programmes, modules, and retreats, but not single inductions) and any control arm; and (3) reported pre‐ and post‐intervention data on ER and/or ED (measured with a validated questionnaire). For the purposes of control arm meta‐regressions, control types were coded as: (a) waitlist controls, involving delayed access to the MBI; (b) treatment‐as‐usual (TAU), defined as ongoing standard clinical care; (c) no intervention, involving neither ongoing standard clinical care nor delayed MBI start; and (d) active controls, defined as alternative interventions incorporating active therapeutic components, deliberately implemented as experimental comparators within the RCT.

### Data selection, extraction, and quality assessment

Five researchers independently screened the titles and abstracts of retrieved studies (TEC, GN, VF, CW, AB) to identify those meeting the inclusion criteria. The same researchers independently assessed the full texts of potentially eligible studies, and the first and senior authors later completed data extraction and study quality assessment. Disagreements were arbitrated by discussion. The study quality of eligible RCTs was assessed using the Cochrane Risk of Bias tool 2 (RoB‐2; Sterne et al., 2019), which indicates bias as follows: (D1) arising from the randomisation process (selection bias); (D2) due to deviations from the intended intervention (adherence); (D3) due to missing outcome data; (D4) in the measurement of outcomes; (D5) in the selection of the reported results; and the overall risk of bias.

### Data synthesis and analysis

Using the R package *esc* (Lüdecke, 2019), the first author (TEC) calculated the Hedge's *g* for each eligible RCT as the standardised mean difference of pre‐ to post‐intervention changes in ER and/or ED between the MBI and control arms. The final datapoint pre‐intervention and the first datapoint post‐intervention were utilised for analyses. All studies included in the meta‐analyses reported means and standard deviations for outcomes at both timepoints in both the intervention and control arms; therefore, no estimation of standard deviations of mean change was required.

Using the R package *metafor* (Viechtbauer, 2010), pooled effect sizes were estimated using random‐effect meta‐analyses, applied separately for each outcome (ER cognitive reappraisal, ER expressive suppression, overall ED, and the multiple ED domains) whenever reported by at least two studies. Cross‐study heterogeneity was tested with Cochran's *Q* and *I* ^*2*^. Funnel plots were used to assess publication bias; we were not able to use any statistical test since no meta‐analysis included more than 10 studies. Meta‐regressions were conducted to investigate potential moderating effects of the control intervention type, mental health condition diagnosis, and risk of bias. All analyses were conducted in R (version 2024.12.0 + 467) by the first author.

## RESULTS

### Characteristics of included studies

**FIGURE 1:**
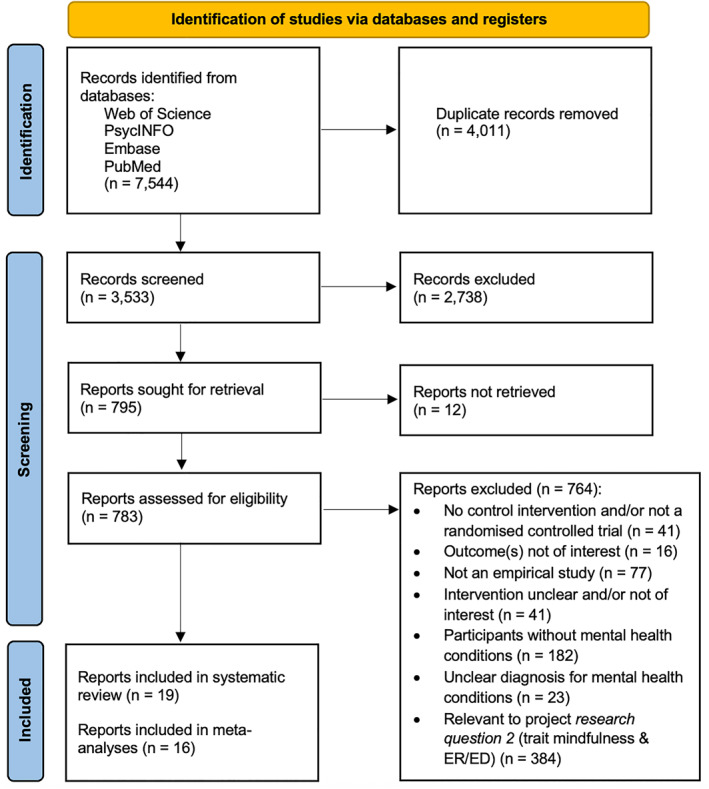
PRISMA flowchart.

Our search returned 7544 records: 4011 were duplicates, and 2738 were deemed irrelevant. After the full‐text screening of the remaining 783 successfully retrieved reports, 19 studies met the inclusion criteria for this review and were included (total *N* = 1,094, 50.03% of whom were randomised to MBIs) (see Figure 1).

Eight studies included data on ER (see Table 1), mostly using the *Emotion Regulation Questionnaire* (ERQ; Gross & John, 2003). One study used the *Cognitive Emotion Regulation Questionnaire* (CERQ) (Garnefski & Kraaij, 2007), which distinguishes between positive and negative cognitive ER (i.e., ‘theoretically more adaptive … [and] less adaptive strategies’, Garnefski & Kraaij, 2007). Another study used the *Emotion Regulation Checklist* (ERC) (Shields & Cicchetti, 1997), which assesses children's ER through ‘adults' perceptions’ (Shields & Cicchetti, 1997). The ERQ comprises two subscales, measuring the use of cognitive reappraisal (ERQ‐CR) and expressive suppression (ERQ‐ES), for which higher scores indicate more frequent use, respectively. 11 studies included data on ED (see Table 2), all using the *Difficulties in Emotion Regulation Scale* (DERS; Gratz & Roemer, 2004), for which higher scores indicate more severe ED. When interpreting effect sizes (i.e., Hedge's *g*) for these outcomes: a positive effect size for cognitive reappraisal (ERQ‐CR) represents an increase in frequency of use; a positive effect size for expressive suppression (ERQ‐ES) represents a reduction in frequency of use; and a negative effect size for emotion dysregulation (DERS—and its subscales) represents a reduction in severity.

**TABLE 1:** Characteristics of studies examining emotion regulation.

Study	Developmental stage	Mental health condition diagnosis	*n*: MBI, control	MBI	Control intervention	Intervention length	Emotion regulation measure	Country	Sample characteristics, %: MBI, control	Summary of findings
Meta‐analysis										
Atta et al. (2024)	Adults (mean: >25 years)	Depressive disorders	60, 60	MBTT (based on Linehan's mindfulness‐based techniques; twice weekly 90‐min group sessions)	TAU	4 weeks	Emotion regulation questionnaire (CR and ES)	Egypt	Female: 30, 30	For both CR and ES, significant within‐group changes for the MBTT arm (increases and decreases, respectively, pre‐ to post‐intervention), and significant between‐group differences in each ER outcome post‐intervention.
Herrmann et al. (2022)	Adults (mean: >25 years)	Various (unspecified, patients admitted to inpatient psychiatric unit for suicidal ideation)	12, 8	MBSI (four, 45‐min individual sessions, over 12 days)	TAU	12 days	Emotion regulation questionnaire (CR and ES)	USA	N/a	No significantly different patterns of change in either CR or ES between the trial arms.
Isham et al. (2022)	Young adults (mean: 18–25 years)	Depressive disorders (in remission)	14, 22	MBSR (weekly two‐hour group sessions, delivered online)	Waitlist	8 weeks	Emotion regulation questionnaire (CR and ES)	UK	Female: 78.6, 74.1	Significantly different patterns of change in CR and ES between the trial arms: significant increases and decreases, respectively, for the MBSR group, though no such significant changes for the control arm.
Lam et al. (2020)	Adults (>18 years, unspecified; mostly >45 years)	Schizophrenia‐spectrum disorder	24, 22	MBPP (based on MBSR; weekly 90‐min group sessions, and between‐session homework/practice)	TAU, and weekly 5‐min telephone check‐in	8 weeks	Emotion regulation questionnaire (CR and ES)	Hong Kong	Female: 75, 77	Significantly different patterns of change in CR between the trial arms (not for ES): Significant increases for the MBPP group post‐intervention, though no such significant change for the control arm. No significant changes in ES in either trial arm.
Vohra et al. (2019)	Children/Adolescents (0–18 years)	Various (unspecified)	42, 39	MBSR (weekly two‐hour group sessions, plus one three‐hour retreat following the eighth session)	TAU	10 weeks	Emotion regulation questionnaire (CR and ES)	Canada	Female: 45.2, 35.9 White: 71.4, 74.4	No significantly different patterns of change in either CR or ES between the trial arms.
Zemestani and Fazeli Nikoo (2020)	Adults (mean: >25 years)	Depressive and anxiety disorders	19, 19	MBCT (weekly two‐hour group sessions, and 30 min daily between‐session homework/practice)	No intervention	8 weeks	Emotion regulation questionnaire (CR and ES)	Iran	Female: 100, 100	Significantly different patterns of change in CR and ES between the trial arms: significant increases and decreases, respectively, for the MBCT group, though no such significant changes for the control arm.
Narrative synthesis										
Elzohairy et al. (2024)	Children/adolescents (mean: 0–18 years)	Attention‐deficit/hyperactivity disorder	26, 24	Mindfulness‐based training programme designed for children with attention‐deficit/hyperactivity disorder (weekly 60‐min sessions, and between‐session homework/practice)	TAU	5 weeks	Emotion regulation checklist	Egypt	Female: 46.2, 58.3	Significantly different patterns of change in ER between the trial arms: significant between‐group differences post‐intervention between the intervention and control arms, with pre‐ to post‐intervention increases and decreases in ER, respectively.
Esmaeili et al. (2018)	Adults (>18 years, unspecified)	Substance use disorders (actively receiving methadone maintenance therapy)	30, 30	MBCT (weekly 90‐min group sessions)	TAU	8 weeks	Cognitive emotion regulation questionnaire (positive and negative)	Iran	Female: 0, 0	Significantly different patterns of change in both positive and negative cognitive ER between trial arms: significant increases and decreases, respectively, for the MBCT group, though no such significant changes in the control arm.

Abbreviations: CR = cognitive reappraisal; ER = emotion regulation; ES = expressive suppression; MBCT = mindfulness‐based cognitive therapy; MBI = mindfulness‐based intervention; MBPP = mindfulness‐based psychoeducation programme; MBSI = mindfulness‐based intervention for suicidal ideation; MBSR = mindfulness‐based stress reduction; MBTT = mindfulness‐based techniques training; TAU = treatment as usual.

**TABLE 2:** Characteristics of studies examining emotion dysregulation.

Study	Developmental stage	Mental health condition diagnosis	*n*: MBI, control	MBI	Control intervention	Length of intervention	Emotion dysregulation measure	Country	Sample characteristics, %: MBI, control	Summary of findings
Meta‐analysis										
Carmona i Farrés et al. (2019)	Adults (mean: >25 years)	Borderline personality disorder	22, 28	DBT‐mindfulness module (weekly 2.5‐h group sessions, and daily between‐session homework/practice)	DBT‐interpersonal effectiveness module (*setup as in the MBI*)	10 weeks	DERS (subscales non‐acceptance, goal‐directedness, impulse, emotional awareness, and clarity)	Spain	Female: 94.3, 85.7	No significantly different patterns of change in the tested DERS subscales between the trial arms.
Costa and Barnhofer (2016)	Adults (mean: >25 years)	Major depressive disorder	19, 18	Mindfulness training (a single 1‐h individual introductory session, followed by 30‐min daily homework/practice)	Guided visual imagery (*setup as in the MBI*)	7 days	DERS (overall only)	UK	Female: 74, 78 White: 74, 61 Black: 16, 39	No significantly different patterns of change in overall ED between the trial arms, though noticeably larger reductions for the MBI arm.
Gawande et al. (2023)	Adults (mean: >25 years)	Various (unspecified)	49, 24	MTPC (weekly 2‐h group sessions, an all‐day retreat, and daily between‐session homework/practice, supported by bi‐weekly engagement calls)	Low‐dose mindfulness comparator	8 weeks	DERS (overall, and all subscales)	USA	Female: 57.1, 62.5 White: 71.4, 83.3 Black: 14.3, 4.2	Significantly different patterns of change in overall ED and DERS subscales goal‐directedness and access to ER: significant decreases in overall ED and issues with goal‐directed behaviour and accessing ER strategies for the MTPC group, though no such significant changes for the control arm.
Gu and Zhu (2023)	Adults (mean: >25 years)	Body dysmorphic disorder	58, 58	MBCT (weekly 90‐min group sessions, and daily between‐session homework/practice)	TAU	8 weeks	DERS (overall only)	China	Female: 77.6, 75.9	Significantly different patterns of change in overall ED: significant decreases in overall ED for the MBCT group, though no such significant changes for the control arm.
Mitchell et al. (2017)	Adults (mean: >25 years)	Attention‐deficit/hyperactivity disorder	11, 9	MAPs for attention‐deficit/hyperactivity disorder (weekly 2.5‐h group sessions, and daily between‐session homework/practice)	Waitlist	8 weeks	DERS (overall, and all subscales)	USA	Female: 54.5, 66.7 White: 81.8, 88.9 Black: 9.1, 11.1	Significantly different patterns of change in overall ED and DERS subscales impulse and access to ER between the trial arms: significant decreases in overall ED and difficulties with impulse control and access to ER for the MAPs group, though no such significant changes for the control arm.
Norouzi et al. (2024)	Adults (>18 years, unspecified)	Major depressive disorder	17, 17	Mindfulness training (twice weekly 45‐min group sessions)	Physical activity (twice weekly group sessions, including static and dynamic aerobic exercise)	8 weeks	DERS (overall only)	Iran	N/a	No significantly different patterns of change in overall ED between the trial arms, though noticeably larger reductions for the MBI arm.
Schanche et al. (2020)	Adults (mean: >25 years)	Depressive disorders (recurrent)	26, 30	MBCT—relapse prevention (weekly 2‐h group sessions, an all‐day silent retreat, and daily between‐session homework/practice)	Waitlist	8 weeks	DERS (only overall for meta‐analysis; all subscales only for narrative synthesis^b^)	Norway	Female: 71, 75.8	Significantly different patterns of change in overall ED and DERS subscales non‐acceptance, access to ER, and clarity: Significant decreases in overall ED and issues with accepting emotional responses, accessing ER strategies, and emotional clarity for the MBCT group, though no such significant changes for the control arm.
Schmidt et al. (2021)	Adults (mean: >25 years)	Borderline personality disorder	50, 52	DBT‐mindfulness module (weekly 2.5‐h group sessions, and daily between‐session homework/practice)	DBT‐interpersonal effectiveness module (*setup as in the MBI*)	10 weeks	DERS (overall only)	Spain	Female: 92.2, 92.3	No significantly different patterns of change in overall ED between the trial arms, though noticeably larger reductions for the DBT‐mindfulness arm.
Spinhoven et al. (2022)	Adults (>18 years, unspecified)	Anxiety disorders	49, 62	MBCT (weekly 2‐h group sessions, and daily between‐session homework/practice)	CBT‐RP (*setup as in the MBI*)	8 weeks	DERS (overall, and all subscales)	the Netherlands	Female: 56.5, 59.5 White: 88.7, 81.1	Different patterns of change in overall ED^a^ between trial arms: significant and large reductions in ED for the MBCT group, though no such significant changes for the control arm.
Weintraub et al. (2023)	Children/Adolescents (13–17 years)	Mood disorders	29, 19	MBCT (weekly 90‐min group sessions delivered online, and between‐session homework/practice encouraged)	CBT (weekly 90‐min group telehealth (Zoom) sessions, and between‐session homework/practice encouraged)	9 weeks	DERS (overall, and all subscales)	USA	Not specified	N/a^b^
Narrative synthesis										
Hamidian et al. (2016) ^c^	Adults (mean: >25 years)	Depressive disorders	22, 22	MBCT (weekly 2–2.5‐h group sessions, and daily between‐session homework/practice)	TAU	8 weeks	DERS (overall, and all subscales)	Iran	N/a	Significantly different patterns of change in overall ED and DERS subscales non‐acceptance, impulse, and access to ER: significant decreases in overall ED and issues with accepting emotion responses, impulse control, and accessing ER strategies for the MBCT group, though no such significant changes for the control arm.

Abbreviations: CBT‐RP = cognitive behavioural therapy—relapse prevention; DBT = dialectical behaviour therapy; DERS = Difficulties in Emotion Regulation Scale; ED = emotion dysregulation; ER = emotion regulation; MAPs = mindful awareness practices; MBCT = mindfulness‐based cognitive therapy; MBI = mindfulness‐based intervention; MBSR = mindfulness‐based stress reduction; MTPC = mindfulness training for primary care; TAU = treatment as usual.

^a^ In the present study, data for the DERS subscales were received via direct request from the first author, which were inputted for computation in the meta‐analysis. Hence, it was not appropriate to narratively discuss the findings for the DERS subscales.

^b^ Data from this study for only participants with mental health conditions were received via direct request from the first author—inputted for computation in the meta‐analysis—since their publication included people without mental health conditions, too. Hence, it was not appropriate to narratively discuss their published, general findings for the present study.

^c^ Results of ANCOVA analyses reported on the DERS subscales, for which data were requested for the present study, though the data were not received in time for our analyses. Thus, we discuss the findings concerning the DERS subscales only narratively.

Three studies could not be included in the meta‐analyses. First, Esmaeili et al. (2018) was the sole study to employ the CERQ, which was deemed conceptually as insufficiently coherent with the other included ER measures, as it assesses multiple cognitive ER strategies beyond cognitive reappraisal alone (e.g., self‐blame, rumination, and catastrophizing). Second, Elzohairy et al. (2024) was the sole study to employ the ERC, which was likewise deemed conceptually as insufficiently coherent with either of the other ER measures, as it adopts a broader operationalisation of ER, including domains such as emotional self‐awareness, emotional expressiveness, emotional lability/negativity, emotional activation, and reactivity. Hence, despite the present study utilising Hedge's *g* effects, outcomes derived from the CERQ and the ERC were not included in the meta‐analyses because of their conceptual uniqueness relative to each other and to the ERQ. Third, Hamidian et al. (2016) was the sole study reporting ANCOVA analysis *F*‐values for ED, which were not sufficient for effect sizes to be calculated (authors were contacted for data request but did not reply within the allocated 2‐week timeframe). Therefore, 16 studies were included in the meta‐analyses (total *N* = 988; 50.71% of whom were randomised to MBIs) and 19 studies in the narrative synthesis.

Sixteen studies included only adults (>18 years), and three studies included only children and adolescents (<18 years). The studies included eight mental health condition diagnoses: mood disorders (*k* = 9), samples that were heterogenous in relation to diagnosis (i.e., transdiagnostic) (*k* = 3), attention‐deficit/hyperactivity disorder (*k* = 2), borderline personality disorder (BPD) (*k* = 2), and (each of the following *k* = 1) schizophrenia spectrum disorder, substance use disorder (participants were actively receiving methadone maintenance therapy from “outpatient drug addiction treatment (methadone) clinics”—Esmaeili et al., 2018), and body dysmorphic disorder. Across the studies that reported participant genders (*k* = 15), 63.48% and 64.78% of those randomised to the MBI and control arms, respectively, were female. Studies were conducted in Europe (*k* = 8), Asia (*k* = 6), North America (*k* = 3), and Africa (*k* = 2).

Risk of bias was rated low for 14.52% of trials (21.57% of studies included in the meta‐analysis) and high for 52.69% of trials (11.76% of studies included in the meta‐analysis), whilst there were some concerns for 32.80% of trials (66.67% of studies included in the meta‐analysis) (See Supporting Information S1: Figure S1 for RoB‐2 assessment summaries for each study).

### Meta‐analysis: Effects of MBIs on emotion regulation

**FIGURE 2:**
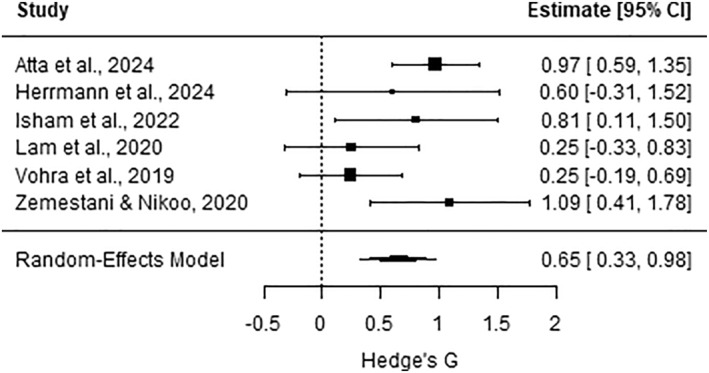
Forest plot of effect sizes for studies examining the effects of mindfulness‐based interventions versus control interventions on ER (cognitive reappraisal) transdiagnostically. Cochrane's *Q*_*5*_ = 9.63, *p* = 0.087, *I* ^*2*^ = 48.40%. The positive effect size for cognitive reappraisal represents a reduction in the frequency at which this ER strategy is utilised. ER, emotion regulation.

MBIs resulted in significantly greater cognitive reappraisal use than control interventions, with a medium‐to‐large effect size (see Table 3 and Figure 2). No significant differences between MBI types were detected (*QM*_*2*_ = 1.34, *p* = 0.521). No significant heterogeneity was found (Cochrane's *Q*_*5*_ = 9.63, *p* = 0.087, *I* ^*2*^ = 48.40%). Despite the small number of effects, evaluation of the funnel plot indicates a low level of publication bias because the plot is broadly symmetrical, and the effect sizes are spread evenly across each side of the pooled effect size (see Supporting Information S1: Figure S2).

**TABLE 3:** Results of meta‐analyses examining the effects of mindfulness‐based interventions versus control interventions on emotion regulation (cognitive reappraisal and expressive suppression) and emotion dysregulation (overall and domains).

Outcome	*k*	Hedge's *g*	SE	95% CI	*p*
Cognitive reappraisal	6	0.65	0.17	[0.33; 0.98]	<0.001*
Expressive suppression	6	−0.25	0.35	[−0.94; 0.45]	0.487
Overall emotion dysregulation	9	−0.54	0.09	[−0.71; −0.36]	<0.001*
Emotion dysregulation domains (DERS subscales)					
Non‐acceptance	5	−0.23	0.15	[−0.52; 0.06]	0.119
Goal‐directedness	5	−0.51	0.12	[−0.75; −0.27]	<0.001*
Impulsivity	5	−0.33	0.12	[−0.56; −0.09]	0.006*
Emotional awareness	5	−0.07	0.12	[−0.30; 0.16]	0.553
Access to emotion regulation	4	−0.54	0.14	[−0.83; −0.26]	<0.001*
Emotional clarity	5	−0.11	0.12	[−0.34; 0.12]	0.361

*Note*: *Indicates a statistically significant result, per the reported *p*‐value.

Abbreviation: DERS = Difficulties in Emotion Regulation Scale.

**FIGURE 3:**
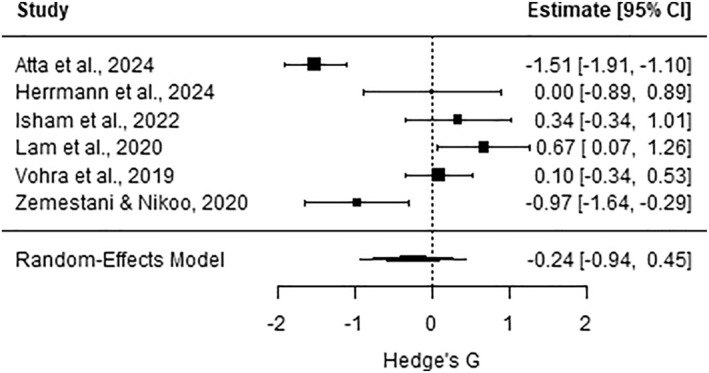
Forest plot of effect sizes for studies examining the effects of mindfulness‐based interventions versus control interventions on ER (expressive suppression) transdiagnostically. Cochrane's *Q*_*5*_ = 53.94, *p* = <0.001, *I* ^*2*^ = 88.53%. The negative effect size for expressive suppression represents a reduction in the frequency at which this ER strategy is utilised. ER, emotion regulation.

Using the same sample, MBIs resulted in lower expressive suppression use than control interventions, with a small, nonsignificant effect size (see Table 3 and Figure 3). No significant differences between MBI types were detected (*QM*_*2*_ = 1.05, *p* = 0.592). Significant heterogeneity was found (Cochrane's *Q*_*5*_ = 53.94, *p* = <0.001, *I* ^*2*^ = 88.53%), though visual inspection shows that the majority of confidence intervals were overlapping. Despite the small number of effects, evaluation of the funnel plot indicates a low level of publication bias because the plot is broadly symmetrical, and the effect sizes are spread evenly across each side of the pooled effect size (see Supporting Information S1: Figure S3).

### Meta‐analysis: Effects of MBIs on emotion dysregulation

**FIGURE 4:**
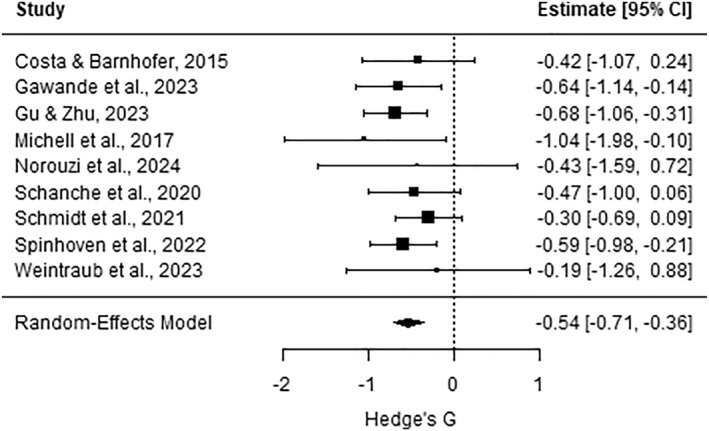
Forest plot of effect sizes for studies examining the effects of mindfulness‐based interventions versus control interventions on overall emotion dysregulation transdiagnostically. Cochrane's *Q*_*7*_ = 3.64, *p* = 0.820, *I* ^*2*^ = 0%. The negative effect size for overall ED represents a reduction in the severity of ED. ED, emotion dysregulation.

MBIs resulted in significantly greater reductions in overall ED (i.e., DERS total scores) than control interventions, with a medium effect size (see Table 3 and Figure 4). No significant differences between MBI types were detected (*QM*_*1*_ = 0.40, *p* = 0.525). No studies used MBSR. No significant heterogeneity was found (Cochrane's *Q*_*8*_ = 4.06, *p* = 0.852, *I* ^*2*^ = 0%). Despite the small number of effects, evaluation of the funnel plot indicates a low level of publication bias because the plot is broadly symmetrical, and results are spread evenly across each side of the pooled effect size (see Supporting Information S1: Figure S4).

MBIs also resulted in significantly greater reductions across several ED domains (DERS subscales) than control interventions. These were: issues engaging in goal‐directed cognitions and behaviour during emotional experience (DERS Goal‐directedness; medium effect; Cochrane's *Q*_*4*_ = 4.07, *p* = 0.397, *I* ^*2*^ = 4.21%), issues engaging in deliberate and/or considered behaviour during emotional experience (DERS Impulsivity; small effect; Cochrane's *Q*_*4*_ = 6.66, *p* = 0.155, *I* ^*2*^ = 0%), and issues accessing ER strategies during emotional experience (DERS Access to ER; medium‐to‐large effect; Cochrane's *Q*_*3*_ = 3.59, *p* = 0.310, *I* ^*2*^ = 11.83%). No significant differences were found between MBIs and control interventions in subscales Non‐acceptance (Cochrane's *Q*_*4*_ = 5.46, *p* = 0.243, *I* ^*2*^ = 31.02%), Emotional Awareness (Cochrane's *Q*_*4*_ = 1.89, *p* = 0.756, *I* ^*2*^ = 0%), or Emotional Clarity (Cochrane's *Q*_*4*_ = 3.51, *p* = 0.476, *I* ^*2*^ = 0%), for which the former had a small‐to‐medium effect and the latter two had negligible effects.

See Table 3 for the full statistical output and Figures S5–S10 in Supporting Information S1: Appendix S3 for the forest plots of effect sizes for each ED domain. Additionally, for the ED domains, we decided not to test heterogeneity or evaluate publication bias due to each meta‐analysis including five or fewer effect sizes. With such limited data, doing so would likely have yielded results of low reliability and validity, thus lacking interpretability (IntHout et al., 2015; van Aert et al., 2019).

### Meta‐regression: Control intervention

The statistical output for all meta‐regressions can be seen in Supporting Information S1: Table S1. A mixed‐effects meta‐regression using effect coding was conducted to assess whether the control type (i.e., no intervention, TAU, or waitlist; no studies measuring cognitive reappraisal used an active control) moderated the effect size of MBIs on cognitive reappraisal use. The moderating effect was nonsignificant. The model did not account for any of the heterogeneity in effect sizes, though residual heterogeneity was large and significant.

Another mixed‐effects meta‐regression using effect coding was conducted to assess whether the control type (i.e., no‐intervention, TAU, or waitlist; no studies measuring expressive suppression used an active control) moderated the effect size of MBIs on expressive suppression use. The moderating effect was nonsignificant. The model did not account for any of the heterogeneity in effect sizes, though residual heterogeneity was moderate and significant.

Another mixed‐effects meta‐regression using effect coding was conducted to assess whether the control type (i.e., TAU, waitlist, or other active interventions; no studies measuring ED used a no‐intervention control) moderated the effect size of MBIs on overall ED. The moderating effect was nonsignificant. The model did not account for any of the heterogeneity in effect sizes, and residual heterogeneity was negligible and nonsignificant.

### Meta‐regression: Mental health condition diagnosis

A mixed‐effects meta‐regression was conducted to assess whether mental health condition diagnosis moderated the effect of MBIs on cognitive reappraisal use. The moderating effect was approaching significance. Further, the model accounted for all of the between‐study variance, and the residual heterogeneity was negligible and nonsignificant.

Another mixed‐effects meta‐regression was conducted to assess whether mental health condition diagnosis moderated the effect of MBIs on expressive suppression use. The moderating effect was nonsignificant. Further, the model accounted for none of the between‐study variance, though the residual heterogeneity was considerable and significant.

A mixed‐effects meta‐regression was also conducted to assess whether mental health condition diagnosis moderated the effect of MBIs on overall ED. The moderating effect was nonsignificant. Further, the model accounted for none of the between‐study variance, and the residual heterogeneity was negligible and nonsignificant.

### Meta‐regression: RoB‐2 scores

Mixed‐effects meta‐regressions were conducted to assess whether RoB‐2 ratings moderated the effects of MBIs on cognitive reappraisal use, expressive suppression use, and overall ED. All significant differences reported for the RoB‐2 categories are based on the meta‐regression coefficients (*β*), representing the difference in effect size relative to the reference category (‘Low’ risk of bias), rather than within‐group significance.

For cognitive reappraisal use, a significant moderating effect was found. There was no residual heterogeneity estimated, and no observed inconsistency across studies; the model accounted for all between‐study variance. ‘Low’ risk of bias studies (the intercept) yielded a nonsignificant and small increase (Hedge's *g* = 0.32, SE = 0.20, 95% CI = [−0.08; 0.71], *p* = 0.118). Estimated differences for studies with ‘Some concerns’ risk of bias were not significant (*β* = 0.17, 95% CI = [−0.43; 0.76], *p* = 0.585), though for studies with ‘High’ risk of bias, estimated differences were significant (*β*, = 0.69, 95% CI = [0.17; 1.20], *p* = 0.009).

For expressive suppression use, a significant moderating effect was found. There was no residual heterogeneity estimated, and no observed inconsistency across studies; the model accounted for all the between‐study variance. ‘Low’ risk of bias studies (the intercept) yielded a nonsignificant and negligible increase (Hedge's *g* = 0.08, SE = 0.20, 95% CI = [−0.32; 0.47], *p* = 0.700). Estimated differences for studies with ‘Some concerns’ risk of bias were not significant (*β* = 0.45, 95% CI = [−0.15; 1.04], *p* = 0.140), though for studies with ‘High’ risk of bias, estimated differences were significant (*β*, = −1.44, 95% CI = [−1.96; −0.92], *p* = <0.001).

For overall ED, no significant moderating effect was found. There was no residual heterogeneity estimated, and no observed inconsistency across studies; the model accounted for none of the between‐study variance. ‘Low’ risk of bias studies (the intercept) yielded a significant, moderate‐to‐large reduction in overall ED (Hedge's *g* = −0.64, SE = 0.26, 95% CI = [−1.14; −0.14], *p* = 0.012). Estimated differences in effect size for studies rated as ‘Some concerns’ (*β* = 0.05, 95% CI = [−0.49; 0.60], *p* = 0.848) or ‘High’ (*β* = 0.34, 95% CI = [−0.29; 0.96], *p* = 0.291) risk of bias were not significant.

## DISCUSSION

To our knowledge, this is the first systematic review and meta‐analysis of RCTs examining the effects of MBIs on ER and ED in people with mental health conditions. 19 RCTs were included (eight concerning ER and 11 concerning ED), with 16 in the meta‐analysis (total *N =* 988), indicating the clinical utility of MBIs as a transdiagnostic approach to improving ER and ED. Overall, we found significantly increased cognitive reappraisal, reduced overall ED, and reduced the ED domains concerning goal‐directedness, impulsivity, and accessing ER strategies following MBIs compared to control interventions.

MBIs significantly increased cognitive reappraisal compared to control interventions, with a moderate‐to‐large effect. Cognitive reappraisal, an ER strategy which involves reframing stimuli to regulate the associated emotion, is linked to improved psychological outcomes across disorders (Gross, 2015; Troy et al., 2018). No significant heterogeneity was detected, indicating consistency across studies, and neither was substantial publication bias detected, indicating that selective reporting was not present. In contrast, MBIs had no significant effect on expressive suppression compared to control interventions. Further, significant heterogeneity was detected, indicating that findings on expressive suppression were inconsistent, though no substantial publication bias was detected, indicating that selective reporting was not present. Notably, the outlying effect of Atta et al. (2024)—which warrants mention—may reflect unusually high intervention intensity and rigour, including extensive preparation, particularly small groups, twice‐weekly sessions, and the integration of Dialectical Behaviour Therapy (DBT) principles, which have been highlighted as potentially beneficial for improving ER issues and ED (Easdale‐Cheele et al., 2024).

These differential effects are consistent with existing literature, whereby mindfulness and cognitive reappraisal are significantly positively correlated, whereas no such correlation is found between mindfulness and expressive suppression (Zhou et al., 2023). This may reflect the adaptive value of the respective strategies in clinical populations. Cognitive reappraisal is generally adaptive across psychopathology severity and contexts, likely due to its antecedent‐focused nature (i.e., construing stimuli in a way that changes the emotional effects) (Gross, 2015; Gross & John, 2003). Although often labelled maladaptive, expressive suppression can also be adaptive, especially in those with severe psychopathology. It may reduce distress and avoid negative social consequences linked to overt and/or intense emotional expression (Gross, 2002, 2015; Gross & John, 2003). Thus, it is plausible that individuals with mental health conditions continue to use suppression, even after MBIs, perhaps until the psychopathology severity reduces and emotional expression becomes less intense and more manageable.

Further, a cognitive‐oriented view suggests that MBIs lend themselves more readily to enhancing cognitive reappraisal than reducing expressive suppression. This may be because MBIs usually improve cognitive skills, such as executive or cognitive control (Teper & Inzlicht, 2013), attentional deployment (Malinowski, 2013), and meta‐cognition (Jankowski & Holas, 2014), that support reappraisal. Conversely, MBIs usually do not target emotional expression directly, but instead simply promote emotional acceptance, which may further aid reappraisal or even function as a reappraisal strategy itself (Roemer et al., 2015).

MBIs significantly reduced overall ED compared to control interventions, with a moderate effect. Significant reductions followed MBIs for several domains of ED, for issues with goal‐directed behaviour when experiencing emotion, impulsivity control relating to emotional responses, and access to ER strategies, each with small‐to‐moderate effects. Concerning overall ED, no significant heterogeneity was detected, indicating consistency across studies, and neither was substantial publication bias detected, indicating that selective reporting was not present.

These findings are consistent with existing literature highlighting psychological and neurobiological overlaps between mindfulness, MBI mechanisms, and ED (Calderone et al., 2024; Gu et al., 2015; Iani et al., 2019; Menefee et al., 2022; Shapero et al., 2018). Improvements in acceptance and non‐reactivity, core mechanisms cultivated in MBIs, correspond with reductions in impulsivity, issues with goal‐directedness, and issues accessing ER strategies (Gu et al., 2015; Iani et al., 2019). These reductions also align with enhanced executive functioning (e.g., attention regulation, cognitive control, and response management) supported by structural and functional improvements in the PFC and ACC (Calderone et al., 2024; Young et al., 2018) typically observed after MBIs, supporting more deliberate approaches to emotional experiences.

Interestingly, MBIs showed no significant effect on the ED domains of emotional non‐acceptance, awareness, or clarity, despite these prescriptively being core targets of MBIs, crucially emphasising acceptance, present moment awareness, and experiential clarity (Guendelman et al., 2017). A possible explanation is a lag in *self‐perception* (Bem, 1972), whereby individuals infer their skills by observing their processes over time. Since the present analyses used the earliest post‐intervention timepoints—immediately upon completion for all included studies—participants may not yet have inferred and thus not reported any changes in these domains. Alternatively, MBIs may foster emotional acceptance, awareness, and clarity *experientially* rather than analytically, which may not align with how these constructs are conceptualised and measured by the DERS (Gratz & Roemer, 2004). Therefore, changes in these ED domains may not have been fully captured.

The risk of bias moderated ER outcomes of cognitive reappraisal and expressive suppression, with stronger effects in lower‐quality studies. ED outcomes appeared more robust, with effects not moderated by the risk of bias, suggesting that MBI‐related improvements in ED are consistent across variations in methodological quality.

Neither the type of control intervention nor the mental health condition diagnosis quantitatively moderated outcomes, highlighting the transdiagnostic potential of MBIs. However, the studies including samples that were heterogeneous in relation to diagnosis (i.e., transdiagnostic, mixed diagnosis samples) showed nonsignificant effects on ER, though significant ED reductions. A potential explanation is that mixed diagnosis samples would permit including comorbid diagnoses, whereas included studies with specific diagnoses mostly excluded participants with comorbid mental health conditions. Therefore, the complexity and baseline levels of psychopathology might have been more severe and convoluted in mixed diagnosis samples, limiting ER improvements, though retaining scope for ED reductions.

Additionally, the two studies including participants diagnosed with BPD, both measuring ED, found nonsignificant effects, indicating less clinical relevance for MBIs to this condition. BPD tends to be characterised by comparatively considerable impairment, including considerable ED, requiring comprehensive, highly structured treatment, such as full programmes of DBT (Leichsenring et al., 2024). Therefore, a potential explanation for this finding is that MBIs alone lack the intensity and specificity required to have a meaningful effect on ED in BPD.

Our systematic review with meta‐analyses suggests that MBIs are promising transdiagnostic treatments that improve cognitive reappraisal and reduce overall ED. Group‐based formats, like MBCT and MBSR, are scalable and well‐suited for limited‐resource mental healthcare systems, such as the NHS. Their comparatively lower training and expertise demands (Zhang et al., 2021) and alignment with *positive psychology* approaches (Allen et al., 2021), which involve promoting long‐term psychological resilience through the cultivation of positive qualities (e.g., eudaimonic and hedonic enhancement), further support their suitability. Unlike short‐term or comparably inconsequential treatments, such as psychopharmacological options, which typically target acute symptoms without fundamental psychological change, MBIs may reduce long‐term service demand by addressing both acute clinical needs and fostering long‐term mental health sustainability, both relevant to ER and ED. Future research could develop and test integrated clinical‐MBI protocols optimised for ER and ED across conditions.

These findings should be interpreted in light of some limitations. First, significant heterogeneity in expressive suppression outcomes indicates variability in effects that may reflect individual differences or intervention‐specific factors (Imrey, 2020), such that our interpretations concerning the effects of MBIs on expressive suppression—and its relationship to cognitive reappraisal—are complicated and seem undermined. Second, the moderating effect of study quality for ER outcomes indicates potential inflation of effects in lower‐quality RCTs. Rigorous RCTs are needed to clarify the present findings. Third, the present meta‐regressions were underpowered due to the low number of effects (i.e., fewer than 10), thus limiting the reliability and interpretability of those analyses (Geissbühler et al., 2021; Higgins et al., 2024). Fourth, only studies using self‐report measures of ER and ED were included in the present study. While these are practical and capture subjective experience (Agako et al., 2022), their validity for the present purposes may be limited since they often reflect stable patterns and thus may not capture post‐intervention changes, potentially due to delayed self‐perception (Bem, 1972). More generally, the introspective nature of these self‐report measures can limit accuracy and validity. Future research is needed to combine self‐report with objective measures of ER and ED, such as behavioural and/or neurophysiological approaches, to strengthen the validity of findings. Lastly, although our search did not restrict participant age, only three studies involved children and/or adolescents. This limits the generalisability of our findings to younger clinical populations, since the presentation and experience of mental health conditions (Caspi et al., 2020) and ER and ED (Helion et al., 2019) can differ across developmental stages. It also precludes meaningful age‐based meta‐regressions and/or subgroup analyses. Future RCTs are needed to address this gap by examining the effects of MBIs on ER and ED in clinical child and adolescent populations.

## CONCLUSION

Our systematic review with meta‐analysis provides evidence supporting the effectiveness of MBIs for improving cognitive reappraisal and reducing ED in people with mental health conditions. Effects were consistent across diagnoses and control types, supporting MBIs' broad clinical relevance. Future RCTs should include younger clinical samples and integrate objective measures of ER and/or ED, and optimise MBI‐based protocol for ER and/or ED. Overall, these findings underscore the promise of MBIs as a transdiagnostic treatment for ER and ED.

## AUTHOR CONTRIBUTIONS

**Thomas Easdale‐Cheele**: Conceptualization; investigation; writing—original draft; methodology; validation; visualization; writing—review and editing; software; formal analysis; data curation; resources; project administration; **George Nash**: Investigation; **Veronika Filobokova**: Investigation; **Chloe Westbury**: Investigation; **Alessio Bellato**: Conceptualization; investigation; writing—review and editing; validation; methodology; supervision.

## CONFLICT OF INTEREST STATEMENT

A.B. declares: (a) funding from the National Institute for Health and Care Research (NIHR208319) and the Academy of Medical Sciences (NGR2\1430), (b) honoraria from the Association for Child and Adolescent Mental Health (ACAMH) for educational activities, (c) honoraria as Joint Editor of JCPP Advances, (d) consultancy honoraria from the Cyprus Research and Innovation Foundation, Swiss National Science Foundation, and Wallenberg Foundation, and (e) travel reimbursements from the ACAMH and the International Brain Research Organisation (IBRO); none of this related to the present project. He is also member of the NHS England ADHD taskforce. The remaining authors have declared that they have no competing or potential conflicts of interest.

## ETHICAL CONSIDERATIONS

An ethics statement is not applicable because this study was a systematic review/meta‐analysis based exclusively on data from published literature.

## Supporting information

Supporting Information S1

## Data Availability

The data that support the findings of this study are available from the corresponding author upon reasonable request.
