# Supplementary material for: The effects of mindfulness‐based interventions on emotion regulation/dysregulation in people with mental health conditions: A systematic review and meta‐analysis
Source: JCPP Adv. 2026 Feb 12;6(3):e70103. doi: 10.1002/jcv2.70103 (PMC13338964; doi:10.1002/jcv2.70103)
Supplement: Supplementary file 1 [file JCV2-6-e70103-s001.docx]

**The effects of mindfulness-based interventions on emotion regulation/dysregulation in people with mental health conditions: A systematic review and meta-analysis**

**Supporting Information**

Appendix S1. PRISMA Checklist

| **Section and**  **Topic** | **Item #** | **Checklist item** | **Location where item is reported** |
| --- | --- | --- | --- |
| **TITLE** | | |  |
| Title | 1 | Identify the report as a systematic review. | Title |
| **ABSTRACT** | | |  |
| Abstract | 2 | See the PRISMA 2020 for Abstracts checklist. | Abstract |
| **INTRODUCTION** | | |  |
| Rationale | 3 | Describe the rationale for the review in the context of existing knowledge. | Introduction |
| Objectives | 4 | Provide an explicit statement of the objective(s) or question(s) the review addresses. | Introduction |
| **METHODS** | | |  |
| Eligibility criteria | 5 | Specify the inclusion and exclusion criteria for the review and how studies were grouped for the syntheses. | Methods,  paragraph “Search Strategy and Selection Criteria” |
| Information sources | 6 | Specify all databases, registers, websites, organisations, reference lists and other sources searched or consulted to identify studies. Specify the date when each source was last searched or consulted. | Methods,  paragraph “Search strategy and Selection Criteria” |
| Search strategy | 7 | Present the full search strategies for all databases, registers and websites, including any filters and limits used. | Appendix S2 |
| Selection process | 8 | Specify the methods used to decide whether a study met the inclusion criteria of the review, including how many reviewers screened each record and each report retrieved, whether they worked independently, and if applicable, details of automation tools used in the process. | Methods,  paragraph “Data Selection, Extraction, and Quality Assessment” |
| Data collection process | 9 | Specify the methods used to collect data from reports, including how many reviewers collected data from each report, whether they worked independently, any processes for obtaining or confirming data from study investigators, and if applicable, details of automation tools used in the process. | Methods,  paragraph “Data Selection, Extraction, and Quality Assessment” |
| Data items | 10a | List and define all outcomes for which data were sought. Specify whether all results that were compatible with each outcome domain in each study were sought (e.g. for all measures, time points, analyses), and if not, the methods used to decide which results to collect. | Methods,  paragraphs “Data Selection, Extraction, and Quality Assessment” and “Data Synthesis and Analysis” |
|  | 10b | List and define all other variables for which data were sought (e.g. participant and intervention | Methods, |

| **Section and**  **Topic** | **Item #** | **Checklist item** | **Location where item is reported** |
| --- | --- | --- | --- |
|  |  | characteristics, funding sources). Describe any assumptions made about any missing or unclear  information. | paragraph “Data Selection,  Extraction, and Quality Assessment”. |
| Study risk of bias assessment | 11 | Specify the methods used to assess risk of bias in the included studies, including details of the tool(s) used, how many reviewers assessed each study and whether they worked independently, and if applicable, details of automation tools used in the process. | Methods,  paragraph “Data Selection, Extraction, and Quality Assessment” and Figure S1. |
| Effect measures | 12 | Specify for each outcome the effect measure(s) (e.g. risk ratio, mean difference) used in the synthesis or presentation of results. | Methods,  paragraph “Data Synthesis and Analysis” |
| Synthesis methods | 13a | Describe the processes used to decide which studies were eligible for each synthesis (e.g. tabulating the study intervention characteristics and comparing against the planned groups for each synthesis (item #5)). | Methods,  paragraph “Data Synthesis and Analysis” |
|  | 13b | Describe any methods required to prepare the data for presentation or synthesis, such as handling of missing summary statistics, or data conversions. | Methods,  paragraph “Data Synthesis and Analysis” |
|  | 13c | Describe any methods used to tabulate or visually display results of individual studies and syntheses. | Methods,  paragraph “Data Synthesis and Analysis” |
|  | 13d | Describe any methods used to synthesize results and provide a rationale for the choice(s). If meta-analysis was performed, describe the model(s), method(s) to identify the presence and extent of statistical heterogeneity, and software package(s) used. | Methods,  paragraph “Data Synthesis and Analysis” |
|  | 13e | Describe any methods used to explore possible causes of heterogeneity among study results (e.g. subgroup analysis, meta-regression). | Methods,  paragraph “Data Synthesis and Analysis” |
|  | 13f | Describe any sensitivity analyses conducted to assess robustness of the synthesized results. | N/a. No sensitivity analyses were conducted. |
| Reporting bias assessment | 14 | Describe any methods used to assess risk of bias due to missing results in a synthesis (arising from reporting biases). | Methods,  paragraph “Data Selection, Extraction, and Quality Assessment” and Figure S1. |
| Certainty assessment | 15 | Describe any methods used to assess certainty (or confidence) in the body of evidence for an outcome. | N/a |
| **RESULTS** | | |  |

| **Section and**  **Topic** | **Item #** | **Checklist item** | **Location where item is reported** |
| --- | --- | --- | --- |
| Study selection | 16a | Describe the results of the search and selection process, from the number of records identified in the search to the number of studies included in the review, ideally using a flow diagram. | Results and Figure 1 |
|  | 16b | Cite studies that might appear to meet the inclusion criteria, but which were excluded, and explain why they were excluded. | Results, paragraph “Characteristics of Included Studies” |
| Study characteristi cs | 17 | Cite each included study and present its characteristics. | Table 2 |
| Risk of bias in studies | 18 | Present assessments of risk of bias for each included study. | Figure S1 |
| Results of individual studies | 19 | For all outcomes, present, for each study: (a) summary statistics for each group (where appropriate) and (b) an effect estimate and its precision (e.g. confidence/credible interval), ideally using structured tables or plots. | Tables 1, 2, and 3; Figures 2, 3, and 4; and Appendix S3 |
| Results of syntheses | 20a | For each synthesis, briefly summarise the characteristics and risk of bias among contributing studies. | Figure S1 |
|  | 20b | Present results of all statistical syntheses conducted. If meta-analysis was done, present for each the summary estimate and its precision (e.g. confidence/credible interval) and measures of statistical heterogeneity. If comparing groups, describe the direction of the effect. | Results and Table 3. |
|  | 20c | Present results of all investigations of possible causes of heterogeneity among study results. | Results |
|  | 20d | Present results of all sensitivity analyses conducted to assess the robustness of the synthesized results. | N/a |
| Reporting biases | 21 | Present assessments of risk of bias due to missing results (arising from reporting biases) for each synthesis assessed. | Figure S1 |
| Certainty of evidence | 22 | Present assessments of certainty (or confidence) in the body of evidence for each outcome assessed. | N/a |
| **DISCUSSION** | | |  |
| Discussion | 23a | Provide a general interpretation of the results in the context of other evidence. | Discussion |
|  | 23b | Discuss any limitations of the evidence included in the review. | Discussion |
|  | 23c | Discuss any limitations of the review processes used. | Discussion |
|  | 23d | Discuss implications of the results for practice, policy, and future research. | Discussion |
| **OTHER INFORMATION** | | |  |
| Registration and protocol | 24a | Provide registration information for the review, including register name and registration number, or state that the review was not registered. | Abstract, Methods |
|  | 24b | Indicate where the review protocol can be accessed, or state that a protocol was not prepared. | Abstract, Methods |

| **Section and**  **Topic** | **Item #** | **Checklist item** | **Location where item is reported** |
| --- | --- | --- | --- |
|  | 24c | Describe and explain any amendments to information provided at registration or in the protocol. | N/a |
| Support | 25 | Describe sources of financial or non-financial support for the review, and the role of the funders or sponsors in the review. | None |
| Competing interests | 26 | Declare any competing interests of review authors. | Conflicts of interest declared |
| Availability of data, code and other materials | 27 | Report which of the following are publicly available and where they can be found: template data collection forms; data extracted from included studies; data used for all analyses; analytic code; any other materials used in the review. | Methods,  paragraphs “Data Selection, Extraction, and Quality Assessment” and “Data Synthesis and Analysis” |

*From:* Page MJ, McKenzie JE, Bossuyt PM, Boutron I, Hoffmann TC, Mulrow CD, et al. The PRISMA 2020 statement: an updated guideline for reporting systematic reviews. BMJ 2021;372:n71. doi: 10.1136/bmj.n71

Appendix S2. Search strategy

Last search: 04/07/2025

Databases searched: Web of Science; PsycINFO; Embase; PubMed. No language or time restrictions were applied.

(mindful* [tiab] OR meditat* [tiab]) AND (“emotion regulation” [tiab] OR “emotional regulation” [tiab] OR “emotion dysregulation” [tiab] OR “emotional dysregulation” [tiab] OR “emotion self-regulation” [tiab] OR “emotional self-regulation” [tiab] OR “irritability” [tiab] OR “emotional lability” [tiab] OR “mood regulation” [tiab] OR “mood dysregulation” [tiab] OR “affect regulation” [tiab] OR “affect dysregulation” [tiab])

Figure S1. RoB-2 assessments for individual studies

| Study | D1 | | D2 | | D3 | | D4 | | D5 | | Overall | |
| --- | --- | --- | --- | --- | --- | --- | --- | --- | --- | --- | --- | --- |
| Meta-analysis | | | | | | | | | | | | |
| Atta et al., 2024 | |  | |  | |  | |  | |  | |  |
| Carmona i Farrés et al., 2019 | |  | |  | |  | |  | |  | |  |
| Costa & Barnhofer,  2015 | |  | |  | |  | |  | |  | |  |
| Gawande et al., 2023 | |  | |  | |  | |  | |  | |  |
| Gu & Zhu, 2023 | |  | |  | |  | |  | |  | |  |
| Herrmann et al., 2024 | |  | |  | |  | |  | |  | |  |
| Isham et al., 2022 | |  | |  | |  | |  | |  | |  |
| Lam et al., 2020 | |  | |  | |  | |  | |  | |  |
| Michell et al., 2017 | |  | |  | |  | |  | |  | |  |
| Norouzi et al., 2024 | |  | |  | |  | |  | |  | |  |
| Schanche et al., 2020 | |  | |  | |  | |  | |  | |  |
| Schmidt et al., 2021 | |  | |  | |  | |  | |  | |  |
| Spinhoven et al., 2022 | |  | |  | |  | |  | |  | |  |
| Vohra et al., 2019 | |  | |  | |  | |  | |  | |  |
| Weintraub et al., 2023 | |  | |  | |  | |  | |  | |  |
| Zemestani & Nikoo, 2020 | |  | |  | |  | |  | |  | |  |
| Narrative synthesis | | | | | | | | | | | | |
| Elzohairy et al., 2024 | |  | |  | |  | |  | |  | |  |
| Esmaeili et al., 2018 | |  | |  | |  | |  | |  | |  |
| sHamidian et al., 2016 | |  | |  | |  | |  | |  | |  |

Figure S2. Funnel plot of effect sizes for studies examining the effects of mindfulness-based interventions versus control interventions on emotion regulation (cognitive reappraisal)

Figure S3. Funnel plot of effect sizes for studies examining the effects of mindfulness-based interventions versus control interventions on emotion regulation (expressive suppression)

Figure S4. Funnel plot of effect sizes for studies examining the effects of mindfulness-based interventions versus control interventions on overall emotion dysregulation

Appendix S3.

Forest plots of effect sizes for studies examining the effects of mindfulness-based interventions versus control interventions on emotion dysregulation domains (per Difficulties in Emotion Regulation Scale subscales)

*Note.* A negative effect size represents a reduction in the severity of that particular emotion dysregulation domain. Conversely, a positive effect size represents an increase in the severity of that particular emotion dysregulation domain.

Figure S5.

*Nonacceptance of emotional responses subscale*

Figure S6.

*Difficulties engaging in goal-directed behaviour subscale*

Figure S7.

*Impulse control difficulties subscale*

Figure S8.

*Lack of emotional awareness subscale*

Figure S9.

*Limited access to emotional regulation strategies subscale*

Figure S10.

*Lack of emotional clarity subscale*

Table S1. Results of meta-regressions assessing the potential moderators of the effects of mindfulness-based interventions versus control interventions

| **Potential moderator** | **Outcome** | ***k*** | ***QM (df)*** | ***R^2^ (%) τ^2^ I^2^(%) QE* (df)** | | | |
| --- | --- | --- | --- | --- | --- | --- | --- |
| Cognitive reappraisal | | 6 | 1.30 (2) | 0 | 0.81 | 89.95 | 38.16 (3)*** |
| Control intervention  Expressive suppression | | 6 | 0.91 (2) | 0 | 0.84 | 91.92 | 46.55 (3)*** |
| Overall emotion dysregulation | | 9 | 1.06 (2) | 0 | 0 | 0 | 3.00 (6) |
| Cognitive reappraisal | | 6 | 9.46 (4) | 100 | 0 | 0 | 0.17 (1) |
| Mental health condition  Expressive suppression | | 6 | 1.04 (4) | 0 | 1.27 | 95.25 | 21.06 (1)*** |
| Overall emotion dysregulation | | 9 | 4.04 (6) | 0 | 0 | 0 | 0.16 (2) |
| Cognitive reappraisal | | 6 | 7.64 (2)* | 100 | 0 | 0 | 1.99 (3) |
| Risk of bias (RoB-2) Expressive suppression | | 6 | 51.58 (2)*** | 100 | 0 | 0 | 2.36 (3) |
| Overall emotion dysregulation | | 9 | 1.88 (2) | 0 | 0 | 0 | 2.18 (6) |

type

diagnosis

*Note. * p* = <.05, ** *p* = <.01, *** *p* = <.001
